# Maternal lithium use and the risk of adverse pregnancy and neonatal outcomes: a Swedish population-based cohort study

**DOI:** 10.1186/s12916-021-02170-7

**Published:** 2021-12-02

**Authors:** Roxanne Hastie, Stephen Tong, Richard Hiscock, Anthea Lindquist, Linda Lindström, Anna-Karin Wikström, Inger Sundström-Poromaa

**Affiliations:** 1 Mercy Perinatal, Mercy Hospital for Women, Melbourne, Australia; 2 Translational Obstetrics Group, Department of Obstetrics and Gynaecology, University of Melbourne, Melbourne, Australia; 3 Department of Women’s and Children’s Health, Uppsala University, Uppsala, Sweden

**Keywords:** Lithium, Pregnancy, Preterm birth, Bipolar, Perinatal psychiatry

## Abstract

**Background:**

Lithium is prescribed during pregnancy, but there is limited information about pregnancy and neonatal outcomes following in utero exposure. Thus, this study aimed to investigate the associations between lithium use and adverse pregnancy and neonatal outcomes.

**Methods:**

This population-based cohort study examined associations between maternal lithium use and major adverse pregnancy and neonatal outcomes via inverse probability weighted propensity score regression models.

**Results:**

Of 854,017 women included in this study, 434 (0.05%) used lithium during pregnancy. Among pre-specified primary outcomes, lithium use during pregnancy was associated with an increased risk of spontaneous preterm birth (8.7% vs 3.0%; adjusted relative risk [aRR] 2.64 95% CI 1.82, 3.82) and birth of a large for gestational age infant (9.0% vs 3.5%; aRR 2.64 95% CI 1.91, 3.66), but not preeclampsia nor birth of a small for gestational age infant. Among secondary outcomes, lithium use was associated with an increased risk of cardiac malformations (2.1% vs 0.8%; aRR 3.17 95% CI 1.64, 6.13). In an analysis restricted to pregnant women with a diagnosed psychiatric illness (*n*=9552), associations remained between lithium and spontaneous preterm birth, birth of a large for gestational age infant, and cardiovascular malformations; and a positive association with neonatal hypoglycaemia was also found. These associations were also apparent in a further analysis comparing women who continued lithium treatment during pregnancy to those who discontinued prior to pregnancy.

**Conclusions:**

Lithium use during pregnancy is associated with an increased risk of spontaneous preterm birth and other adverse neonatal outcomes. These potential risks must be balanced against the important benefit of treatment and should be used to guide shared decision-making.

**Supplementary Information:**

The online version contains supplementary material available at 10.1186/s12916-021-02170-7.

## Background

Lithium is well-established as an effective first-line treatment for bipolar disorder, a major psychiatric condition affecting 2% of the global population [1]. With an average age of disease onset of 25 years, many women of reproductive age are offered lithium. Deciding whether to continue lithium during pregnancy requires balancing the risk of exposure to the fetus with the benefits of treatment for the mother. The maternal advantages of continuing lithium during pregnancy are clear. Women who stop treatment during pregnancy are almost threefold more likely to experience recurrence or relapse compared to those continuing prophylactic medications [2]. Lithium is also effective in preventing suicide [3]. Thus, discontinuation may put women at risk of self-harm and deterioration of psychosocial and medical well-being.

In contrast, associations between lithium exposure in utero and the risk of adverse pregnancy or neonatal outcomes are unclear. Associations between lithium use and congenital malformations have been reported, including cardiac malformations, which suggest an increased likelihood of malformation [4–6]. Studies exploring potential associations between lithium and adverse pregnancy outcomes have been limited to small observational studies and case reports. Meta-analyses of these have reported an increased likelihood of spontaneous abortion [4] and neonatal readmission to hospital [5].

Better information on the possible impact of lithium administration and adverse pregnancy outcomes could aid clinical decision-making and surveillance in the management of psychiatric illness during and after pregnancy. To address this gap in knowledge, we performed a population-based study examining whether maternal lithium use was associated with adverse pregnancy outcomes.

## Methods

### Study population

This retrospective population-based cohort study obtained data through linkage of 5 Swedish national registers using unique personal identity numbers that are assigned to all Swedish residents. Linked registers included the Swedish Medical Birth Register, the Patient Register, the Prescribed Drug Register, Education Register and Total Population Register. The Medical Birth Register prospectively collects data of over 98% of all births in Sweden and includes demographic data, reproductive history, complications during pregnancy and delivery and neonatal outcomes. The Patient Register includes information on all inpatient care and outpatient clinical services, including psychiatry. All procedures and diagnoses are documented using International Classification of Disease (ICD) diagnostic codes. The Prescribing Drug Register contains data on all prescriptions dispensed in Sweden and includes information on dispensed item, date of dispensing, substance, formulation, package size, dispensed amount and dosage. Linkage between the registers is possible due to the individual identification number given to all citizens in Sweden.

All women giving birth to a liveborn or stillborn child at 22 gestational weeks or later in Sweden from January 2007 to December 2014 were included. Multiple pregnancies were excluded.

### Exposure

Maternal lithium treatment prior and during pregnancy was obtained from the Prescribing Drug Register (ATC code N05AN). Lithium use during pregnancy was defined as a prescription dispensed during pregnancy or the 3 months prior to conception. To identify lithium exposure during this period, date of conception was calculated as date of birth − gestational age in days, with gestational age at birth obtained from the Medical Birth Register and estimated via ultrasound dating during the second trimester of pregnancy.

### Pregnancy and neonatal outcomes

Primary and secondary outcomes of interest were prespecified and selected based on potential impacts of medication exposure during pregnancy and findings from previous studies. The primary outcomes were preeclampsia (ICD-10 codes O14, O15), spontaneous preterm birth before 37 weeks’ gestation and birth of a small for gestational age or large for gestational age infant. Information on onset of birth is routinely recorded in a standardized manner by the delivery ward midwife and is categorized as spontaneous, induced vaginal or caesarean section before onset of labour. Spontaneous onset of birth was defined as a registered spontaneous start or if a diagnosis of preterm premature rupture of the membranes (ICD code O42) was present. Small for gestational age and large for gestational age were defined as a birthweight of more than two standard deviations below or above the mean weight for gestational age according to the Swedish national reference curve [7]. Secondary outcomes were macrosomia (birthweight > 4000 g), hypoglycaemia (ICD code P70), Apgar < 7 at 5 min, perinatal death, any congenital malformation and cardiac malformations: ICD-10 code Q20 congenital malformations of cardiac chambers and connections, Q21 congenital malformations of cardiac septa, Q22 congenital malformations of pulmonary and tricuspid valves, Q23 congenital malformations of aortic and mitral valves, Q24 other congenital malformations of heart, Q25 congenital malformations of great arteries and Q26 congenital malformations of great veins.

### Covariates

Information on maternal age at delivery (</≥35 years), height, weight, smoking at first antenatal visit (yes/no), parity (nulliparous or multiparous), use of assisted reproduction (yes/no) and gestational disorders was obtained from the medical birth register. Body mass index (BMI) was calculated as weight (kg)/height (m^2^) and categorized as (</≥ 30 kg/m^2^. Data on country of birth was obtained from the Total Population Register and categorized as Sweden, other European countries or the rest of the world. Educational attainment was retrieved from the Higher Education Registry and categorized as < 12 years, completion of high school or completion of university. Pre-existing maternal conditions were obtained by ICD codes from the Patient Register: bipolar disorder (F31), schizophrenia (F20–F29), psychosis (F29), pre-gestational hypertension (I10, O10) and diabetes (O240, O241, O244, O249, E10–14).

### Subgroup analyses

To account for the impact of underlying psychiatric illness on perinatal outcomes, we performed two subgroup analyses. Both were planned a priori. First, we compared the same outcomes among women with a diagnosis of bipolar disorder, schizophrenia or psychosis who were either prescribed lithium or not.

Second, we compared women who had used lithium during pregnancy to those who had used lithium prior to pregnancy but not in the 3 months prior to conception or during pregnancy.

### Statistical analysis

A prespecified statistical analysis plan was generated prior to analyses and agreed upon by the authors. Characteristics of the population were described according to lithium use during pregnancy. Lithium users and non-users were compared via bivariate analysis using Pearson’s Chi-squared test for categorical data and Student’s *t* test for continuous variables.

The average treatment effect for lithium-treated women (average treatment effect on the treated) on adverse pregnancy and neonatal outcomes was estimated using Stata’s ‘teffects’ command and presented as relative risk with 95% confidence intervals; within-mother clustering was accounted for in standard errors. Regression models were inverse probability weighted to achieve covariate balance between exposure groups. A propensity score for exposure was calculated for each individual using a logistic regression that included variables considered likely confounders based upon directed acyclic graphs and selected a priori for inclusion in the propensity score models. Included covariates were: maternal age (</> 35 years), body mass index (</> 30 kg/m^2^), smoking status, country of birth, education, parity, maternal psychiatric disorders (schizophrenia and psychosis, with an interaction term between these), other medical conditions (diabetes, hypertension) and antipsychotics (ATC code N05A), central stimulants (ATC-code N06BA), or lamotrigine (ATC code N03AX09) dispensed during or 3 months prior to pregnancy. Second, with the aim of achieving balance between exposed and unexposed groups, the estimated propensity scores were used to weight each subject, with a score of 1/propensity score assigned to the exposed individuals and 1/(1 − propensity score) to the unexposed. Propensity scores were also generated for subgroup analyses, with the following covariates included: maternal age (</> 35 years), body mass index (</> 30 kg/m^2^), smoking status, country of birth, education, parity, medical conditions (diabetes and hypertension) and antipsychotics, neuroleptics or lamotrigine dispensed during or 3 months prior to pregnancy. Propensity scores were checked for extreme values and overlap between exposure groups. The balance of individual covariates before and after inverse probability weighting was also assessed. Weighted standardized differences of less than 0.1 were indicative of covariate balance (raw and weighted standardized differences for included covariates and each outcome are shown in Additional File 1). Modelling only proceeded when there was sufficient overlap of propensity scores and covariate balance following weighting was achieved. Multiple imputation of exposure, outcome and covariate missing data was not performed and those with missing data were not included in the adjusted analysis.

Statistical analysis used StataMP® software (StataCorp. College Station, TX, USA).

## Results

### Participant characteristics

Of the 854,017 women studied, 434 (0.05%) used lithium during pregnancy. Among the whole cohort, the mean maternal age was 30.3 years (standard deviation (SD) 5.3), body mass index was 24.7 (SD 4.6) and most were born in Sweden (77.6%; Table 1). Individual covariate data were missing for 0.7% to 9.9% of the total study population, and only 3.5% of the cohort had more than one missing characteristic.

**Table 1:** Maternal characteristics by lithium use

Characteristic	Total births *n*= 854,017	Lithium exposure	
		No (*n*=853,583)	Yes (*n*=434)
**Age,** (years), mean ±SD	30.3 ± 5.3	30.3 ± 5.3	31.7 ± 5.2
≥35, *n* (%)	187,258 (21.9)	187,123 (21.9)	135 (31.1)
**Body mass index** (kg/m^2^), mean ± SD	24.7± 4.6	24.7 ± 4.63	26.3 ± 4.1
BMI≥30, *n* (%)	99,010 (11.6)	98,930 (11.6)	80 (18.4)
Missing, *n* (%)	59,171 (6.9)	59,128 (6.9)	43 (9.9)
**Parity**, *n* (%)			
Nulliparous	378,644 (44.3)	378,425 (44.3)	219 (50.5)
Multiparous	475,373 (55.7)	475,158 (55.7)	215 (49.5)
**Country of birth**, *n* (%)			
Sweden	662,597 (77.6)	662,206 (77.6)	391 (90.1)
Other European countries	27,917 (3.3)	27,907 (3.3)	10 (2.3)
Rest of the world	163,503 (19.2)	163,470 (19.2)	33 (7.6)
**Smoking,** *n* (%)			
Yes	51,885 (6.1)	51,818 (6.1)	67 (15.4)
Missing	33,208 (3.9)	33,192 (3.9)	16 (3.7)
**Assisted reproduction,** *n* (%)			
Yes	23,886 (2.8)	23,882 (2.8)	4 (0.9)
**Education,** *n* (%)			
University	451,084 (52.8)	450,851 (52.8)	233 (53.7)
High school	234,694 (27.5)	234,585 (27.5)	109 (25.1)
< 12 years	158,452 (18.6)	158,363 (18.6)	89 (20.5)
Missing	9787 (1.2)	9784 (1.2)	3 (0.7)
**Pre-gestational disorders,** *n* (%)			
Bipolar disorder	7603 (0.9)	7198 (0.8)	405 (93.3)
Psychosis	2182 (0.3)	2117 (0.3)	65 (15.0)
Schizophrenia	2470 (2.3)	2398 (0.3)	72 (16.6)
Hypertension	6365 (0.8)	6358 (0.7)	7 (1.6)
Diabetes	6525 (0.8)	6522 (0.8)	3 (0.7)
**Gestational diabetes,** *n* (%)	9810 (1.2)	9803 (1.2)	7 (1.6)
**Gestational age,** weeks	39.8 ± 1.8	39.8 ± 1.8	39.2 ± 2.3
**Birthweight,** grams, mean ± SD	3536 ± 565	3536 ± 565	3500 ± 679

Compared to unexposed women, lithium-exposed women were older, more obese, more frequently nulliparous and smokers, whilst less likely to have conceived via assisted reproduction techniques or be born outside of Sweden. Additionally, women using lithium were more likely to have pre-existing hypertension (Table 1).

### Main outcomes

The incidence of spontaneous preterm birth was higher among women using lithium, compared to those not (8.7% vs 3.0%, crude relative risk (RR) of 2.80 (95% confidence interval [CI] 2.02, 3.88)). After adjusting via inverse probability weighting, lithium use remained associated with a two-fold increased risk of spontaneous preterm birth (adjusted RR 2.64 [95% CI 1.82, 3.82]; Table 2). Of women prescribed lithium and having a spontaneous preterm birth the median gestational age at birth was 35.2 weeks (interquartile range 33.7–36.4).

**Table 2:** Primary outcome compared to whole population

	No lithium ***n***= 853,583	Lithium use ***n***=434		
	*n* (%)	*n* (%)	Relative risk (95% confidence interval)	
			Crude	Adjusted
**Primary outcomes**				
Preeclampsia	24,008 (2.8)	15 (3.5)	1.29 (0.75, 2.02)	1.01 (0.59, 1.73)
Spontaneous preterm birth Missing *n*=723	25,268 (3.0)	36 (8.7)	2.80 (2.02, 3.88)	2.64 (1.82, 3.82)
Small for gestational age Missing *n*=1446	19,794 (2.3)	13 (3.0)	1.29 (0.70, 2.38)	1.05 (0.54, 2.06)
Large for gestational age Missing *n*=1446	29,619 (3.5)	39 (9.0)	2.59 (1.91, 3.51)	2.64 (1.91, 3.66)
**Secondary outcomes**				
Macrosomia Missing *n*=1116	158,461 (18.6)	93 (21.4)	1.01 (0.25, 4.03)	1.05 (0.25, 4.32)
Hypoglycaemia	20,877 (2.5)	24 (5.5)	2.26 (1.54, 3.33)	1.46 (0.89, 2.40)
Five-minute Apgar < 6 Missing *n*=5011	11,221 (1.3)	11 (2.5)	1.92 (1.02, 3.61)	0.92 (0.38, 2.22)
Malformations (all)	29,240 (3.4)	19 (4.4)	1.28 (0.83, 1.98)	1.41 (0.90, 2.23)
Cardiac malformations	6513 (0.8)	9 (2.1)	2.72 (1.43, 5.17)	3.17 (1.64, 6.13)
Perinatal death	3567 (0.5)	2 (0.5)	1.58 (0.39, 6.27)	1.08 (0.15, 7.67)

Adjusted analyses were retrieved via inverse probability weighting with maternal age, body mass index, smoking status, country of birth, education, parity, maternal psychiatric illness (schizophrenia, psychosis) and medical conditions, and the use of antipsychotics, neuroleptics and lamotrigine during pregnancy were included as covariates

Regarding other pre-specified primary outcomes, lithium use was associated with a higher risk of birthing a large for gestational age infant, with a crude relative risk of 2.59 (95% CI 19.2, 3.50; 9.0% vs 3.5%) and an adjusted relative risk of 2.64 (95% CI 1.91, 3.66; 9.0% vs 3.5%) (Table 2). There was no association between lithium use and preeclampsia (3.5% vs 2.8%; aRR 1.01 [95% CI 0.59, 1.73) or birth of a small for gestational age infant (3.0% vs 2.3%; aRR 1.05 [95% CI 0.54, 2.06]).

Among secondary outcomes across the whole population, no associations were found between lithium use and macrosomia (defined as birthweight > 4500 g; 21.4% vs 18.6%; aRR 1.05 [95%CI 0.25, 4.32]), neonatal hypoglycaemia (5.5% vs 2.5%; aRR 1.46 [95% CI 0.89, 2.40]), a 5-min Apgar score < 6 (2.5% vs 1.3%; aRR 0.92 [95% CI 0.38, 2.22]), total malformations (4.4% vs 3.4%; aRR 1.41 [95% CI 0.90, 2.23]) or perinatal death (0.5% vs 0.5%; aRR 1.08 [95% CI 0.15, 7.67]). However, a higher incidence of neonates with cardiac malformations was observed among women who used lithium (2.1% vs 0.8%), with an aRR of 3.17 (95% CI 1.64, 6.13) and of these, the majority (66.6%) were reported as malformations of the cardiac septa (ICD-10 Q21).

### Subgroup analysis

We next compared women using lithium during pregnancy (*n*=434) to those who were prescribed lithium prior to pregnancy, but not during (*n*=871). Women using lithium during pregnancy, compared with those who discontinued lithium prior to pregnancy, had an increased risk of spontaneous preterm birth (8.7% vs 4.4%; aRR 2.08 [95% CI 1.20, 3.59]), birth of a large for gestational age infant (9.0% vs 4.3%; aRR 1.86 [95% CI 1.10, 3.13]), neonatal hypoglycaemia (5.5% vs 2.6%; aRR 2.72 [95% CI 1.47, 5.04]) and cardiac malformations (2.1% vs 0.8%, aRR 2.99 [95% CI 1.10, 8.10]; Table 3).

**Table 3:** Comparison to women using lithium prior to pregnancy but not during

	Lithium prior ***n***=871	Lithium during ***n***=434		
	*n* (%)	*n* (%)	Relative risk (95% confidence interval)	
			Crude	Adjusted
**Primary outcomes**				
Preeclampsia	33 (3.8)	15 (3.5)	0.91 (0.50, 1.67)	0.86 (0.44, 1.70)
Spontaneous preterm birth	37 (4.4)	36 (8.7)	1.95 (1.23, 3.09)	2.08 (1.20, 3.59)
Small for gestational age	31 (3.6)	13 (3.0)	0.84 (0.42, 1.70)	0.91 (0.42, 2.01)
Large for gestational age	37 (4.3)	39 (9.0)	2.12 (1.37, 3.28)	1.86 (1.10, 3.13)
**Secondary outcomes**				
Macrosomia	138 (15.8)	93 (21.4)	0.67 (0.14, 3.30)	0.50 (0.09, 2.68)
Hypoglycaemia	23 (2.6)	24 (5.5)	2.09 (1.20, 3.66)	2.72 (1.47, 5.04)
Five-minute Apgar < 6	16 (1.9)	11 (2.5)	1.37 (0.62, 3.04)	2.25 (0.89, 5.66)
Malformations (all)	25 (2.9)	19 (4.4)	1.52 (0.85, 2.73)	1.47 (0.77, 2.80)
Cardiac malformations	7 (0.8)	9 (2.1)	2.58 (0.97, 6.86)	2.99 (1.10, 8.10)_
Perinatal death	3 (0.3)	2 (0.5)	2.68 (0.45, 15.90)	2.26 (0.21, 24.54)

Adjusted analyses were retrieved via inverse probability weighting with maternal age, body mass index, smoking status, country of birth, education, parity and medical conditions, and the use of antipsychotics, neuroleptics and lamotrigine during pregnancy were included as covariates

We also compared the same outcomes among women diagnosed with a maternal psychiatric illness (bipolar, schizophrenia and psychosis) and either prescribed lithium (*n*=412) or not (*n*=9140). Associations observed were similar to the primary analysis: spontaneous preterm birth remained associated with maternal lithium use (8.0% vs 3.4%; aRR of 2.34 [95% CI 1.55, 3.56]), as was the risk of birthing a large for gestational age infant (9.5% vs 4.1%; aRR 2.28 [95% CI 1.61, 3.23]), but not preeclampsia nor birthing a small for gestational age infant (Table 4). Regarding secondary outcomes, lithium use was associated with an increased risk of neonatal hypoglycaemia (5.3% vs 3.0%; aRR 1.59 [95% CI 1.01, 2.49]) and cardiac malformations (1.9% vs 0.8%; aRR 3.01 [95% CI 1.38, 6.53]).

**Table 4:** Perinatal outcomes among women diagnosed with bipolar disorder, psychosis or schizophrenia

	No lithium ***n***=9140	Lithium use ***n***=412		
	*n* (%)	*n* (%)	Relative risk (95% confidence interval)	
			Crude	Adjusted
Preeclampsia	286 (3.1)	15 (3.6)	1.16 (0.70, 1.94)	0.95 (0.54, 1.64)
Spontaneous preterm birth	314 (3.4)	33 (8.0)	2.33 (1.62, 3.34)	2.34 (1.55, 3.56)
Small for gestational age	235 (2.6)	12 (2.9)	1.13 (0.59, 2.18)	1.05 (0.50, 2.19)
Large for gestational age	374 (4.1)	39 (9.5)	2.32 (1.68, 3.18)	2.28 (1.61, 3.23)
**Secondary outcomes**				
Macrosomia	42 (0.5)	2 (0.5)	1.06 (0.26, 4.35)	1.16 (0.27, 4.96)
Hypoglycaemia	271 (3.0)	22 (5.3)	1.80 (1.18, 2.74)	1.59 (1.01, 2.49)
Five-minute Apgar < 6	175 (1.9)	11 (2.7)	1.07 (0.50, 2.72)	1.07 (0.50, 2.28)
Malformations (all)	309 (3.4)	18 (4.4)	1.29 (0.81, 2.05)	1.35 (0.82, 2.21)
Cardiac malformations	72 (0.8)	8 (1.9)	2.46 (1.20, 5.07)	3.01 (1.38, 6.53)
Perinatal death	40 (0.4)	2 (0.5)	1.69 (0.41, 7.03)	1.01 (0.14, 7.56)

Adjusted analyses were retrieved via inverse probability weighting with maternal age, body mass index, smoking status, country of birth, education, parity and medical conditions, and the use of antipsychotics, neuroleptics and lamotrigine during pregnancy were included as covariates

## Discussion

In this nationwide population-based study lithium taken during or the 3 months prior to pregnancy was associated with an increased risk of spontaneous preterm birth, birth of a large for gestational age infant and cardiac malformations. Importantly, these associations remained in a pre-specified sub-analysis restricted to women with a diagnosis of bipolar disorder, schizophrenia or psychosis; and a further pre-specified analysis examining mothers who used lithium during pregnancy compared to those who took lithium prior to pregnancy but not during. Among these two subgroup analyses, an increased risk of neonatal hypoglycaemia was also observed in addition to the complications observed in the primary analysis. Reassuringly, however, we did not uncover associations between lithium use and preeclampsia, nor birth of a small for gestational age infant. These two complications can reflect placental dysfunction and foetuses that are small for gestational age incur an increased stillbirth risk [8].

In contrast to our results, two meta-analyses’ have reported no association between maternal lithium use and preterm birth [4, 5]. Importantly, both these studies did not differentiate between iatrogenic (where preterm birth was medically indicated) and spontaneous preterm birth, whereas our study focussed on spontaneous preterm birth. The strength of our finding is that spontaneous preterm birth was consistently associated with lithium use when we compared women using lithium to not only the unexposed whole population, but also among women with psychiatric illness and those who ceased lithium prior to pregnancy.

We do note that like our study, the meta-analysis published during 2018 also included Swedish national data. Within the Swedish arm of the previous study, 238 lithium-exposed women were compared to a reference group of 13,407 women diagnosed with a mood disorder. This study investigated several pregnancy and delivery complications as well as neonatal readmission for congenital malformations. In contrast, the present study included 434 lithium-exposed women, who were compared to three reference groups. Additionally, using an a priori statistical analysis plan we focussed on four major pregnancy complications as our primary outcomes. Two of the four, spontaneous preterm birth and large for gestational age infants, were not reported in the prior study. Furthermore, we used a propensity score-based approach to control for important baseline differences between women using lithium and those not.

Previous studies reporting congenital malformations have been conflicting. A meta-analysis published in 2020 concluded lithium was associated with an increased likelihood of congenital malformations overall and specifically, association with cardiac malformations [4]. In the present study, lithium use was not associated with congenital malformations overall, however further analysis revealed an increased risk of cardiac malformations among lithium-exposed women. It is important to note that across all studies, including ours, the absolute risk of malformation is low.

Given large for gestational age infants are at increased risk of neonatal hypoglycaemia [9], identifying both to be associated with lithium use strengthens the plausibility of our findings. Additionally, lithium use has been associated with altered blood glucose levels and hypoglycaemia in adults [10, 11], thus increasing the plausibility of this association in the neonate. Although we did not explore gestational diabetes within the present study, rates were similar in women exposed and unexposed to lithium during pregnancy. Additionally, two previous meta-analyses reported no association between maternal lithium use and gestational diabetes [4, 5]. Thus, the association between lithium and birth of a large for gestational age infant and neonatal hypoglycaemia are unlikely to be attributed to an increased risk of gestational diabetes.

### Strengths and limitations

To our knowledge, our study is the largest cohort from one country to examine perinatal outcomes among women using lithium during pregnancy. Importantly, we were able to perform two planned sub-analyses that corrected for underlying psychiatric illness (which itself has been associated with adverse perinatal outcomes). We also compared those who took lithium prior to, but not during pregnancy versus those who took the drug during pregnancy. The same associations held between lithium and major adverse outcomes remained in all these analyses (preterm birth, birthing a large for gestational age infant and cardiac malformations). Another strength is that we found a likely association with cardiac malformation which has been previously shown [4]. Finally, we used a propensity score inverse probability weighting approach, which balanced covariates that differed between those women using lithium and those not.

There are some limitations to our study. Whilst we pre-specified our analysis and limited our choices for the primary outcomes to major obstetric conditions, four outcomes were investigated. This raises the potential for a type 1 error. Also, whilst the use of inverse probability weighting allowed us to adjust for a number of confounders, it remains plausible that residual confounding through unmeasured covariates may have occurred. Additionally, women with missing covariate data were not included in adjusted analyses. Lastly, medication use was obtained via the Swedish Prescribed Drug Register, which contains data on all dispensed prescriptions but does not provide data confirming that the lithium was consumed. However, this would have been expected to attenuate the strength of associations. Furthermore, future studies investigating the associations between actual lithium use or maternal serum levels and adverse obstetric and long-term offspring health and developmental outcomes are needed.

## Conclusions

The use of lithium during pregnancy is associated with a 2–3-fold increased risk of spontaneous preterm birth, birth of a large for gestational age infant and cardiac malformations. It is important to note that the absolute risk of malformations remained low. Lithium use may also be associated with neonatal hypoglycaemia. Lithium was not associated with an increased risk of preeclampsia or birthing a small for gestational age infant. These findings should be weighed against the need to effectively manage maternal psychiatric illness during pregnancy and should be used to guide shared treatment decision making and appropriate surveillance for those on treatment during pregnancy.

## Supplementary Information

**Additional file 1: Table S1.** Raw and weighted standardized differences for included covariates and each outcome.

## Data Availability

The data that support the findings of this study are available from Statistics Sweden, but restrictions apply to the availability of these data, and so are not publicly available. Data can be accessed via application to Statistics Sweden.

## Abbreviations

ICD: International Classification of Disease
RR: Relative risk
aRR: Adjusted relative risk
95% CI: 95% confidence interval
