# Supplementary material for: Maternal lithium use and the risk of adverse pregnancy and neonatal outcomes: a Swedish population-based cohort study
Source: BMC Med. 2021 Dec 2;19:291. doi: 10.1186/s12916-021-02170-7 (PMC8641220; doi:10.1186/s12916-021-02170-7)
Supplement: Supplementary file 1 — Additional file 1: Table S1. Raw and weighted standardized differences for included covariates and each outcome. [file 12916_2021_2170_MOESM1_ESM.docx]

|  | **Total population**  **Standardized differences** | | **Diagnosis of bipolar, schizophrenia or psychosis** | | **Lithium use prior/during pregnancy** | |
| --- | --- | --- | --- | --- | --- | --- |
|  | **Raw** | **Weighted** | **Raw** | **Weighted** | **Raw** | **Weighted** |
| Schizophrenia | 0.61 | -0.06 | - | - | - | - |
| Psychosis | 0.57 | -0.03 | - | - | - | - |
| Interaction of Schizophrenia psychosis | 0.46 | -0.03 | - | - | - | - |
| Antipsychotics during pregnancy | - | - | 0.63 | <0.01 | 0.51 | <0.01 |
| Central stimulants during pregnancy | 0.27 | -0.05 | 0.08 | <0.01 | -0.07 | 0.03 |
| Lamotrigine during pregnancy | 0.46 | -0.07 | 0.16 | -0.01 | 0.04 | <0.01 |
| Maternal age (>35 year) | 0.21 | 0.01 | 0.27 | -0.01 | 0.12 | <0.01 |
| BMI (30 kg/m) | 0.22 | -0.01 | 0.03 | <0.01 | 0.01 | -0.03 |
| Parity | -0.12 | <0.01 | -0.12 | <0.01 | -0.03 | -0.04 |
| Education | 0.03 | 0.03 | -0.36 | <0.01 | -0.26 | <0.01 |
| Country of birth | -0.28 | -0.01 | -0.14 | <0.01 | 0.03 | -0.03 |
| Smoking | 0.33 | -0.02 | -0.07 | <0.001 | -0.04 | <0.01 |
| Type 1 & 2 diabetes mellitus | -0.001 | 0.001 | -0.01 | -0.01 | -0.01 | <0.01 |
| Chronic hypertension | 0.09 | -0.01 | 0.04 | <0.01 | 0.08 | <0.01 |

**Table S1: Covariate balance**
